# Supplementary material for: Two mitochondrial DNA polymorphisms modulate cardiolipin binding and lead to synthetic lethality
Source: Nat Commun. 2024 Jan 19;15:611. doi: 10.1038/s41467-024-44964-2 (PMC10799063; doi:10.1038/s41467-024-44964-2)
Supplement: Supplementary file 1 — Supplementary information [file 41467_2024_44964_MOESM1_ESM.pdf]

Supplementary files

<u>Title:</u> Two mitochondrial DNA polymorphisms modulate cardiolipin binding and lead to synthetic lethality

Authors: Ason CY Chiang[1,2,3], Jan Jezek[2,3,4], Peiqiang Mu[2,3,5], Ying Di[2,3,6], Anna Klucnika[2,3,7], Martin Jabůrek[8], Petr Ježek[8], Hansong Ma[1,2,3 *]

This PDF file contains
-Supplementary Figures 1-5
-Supplementary Tables 1-4

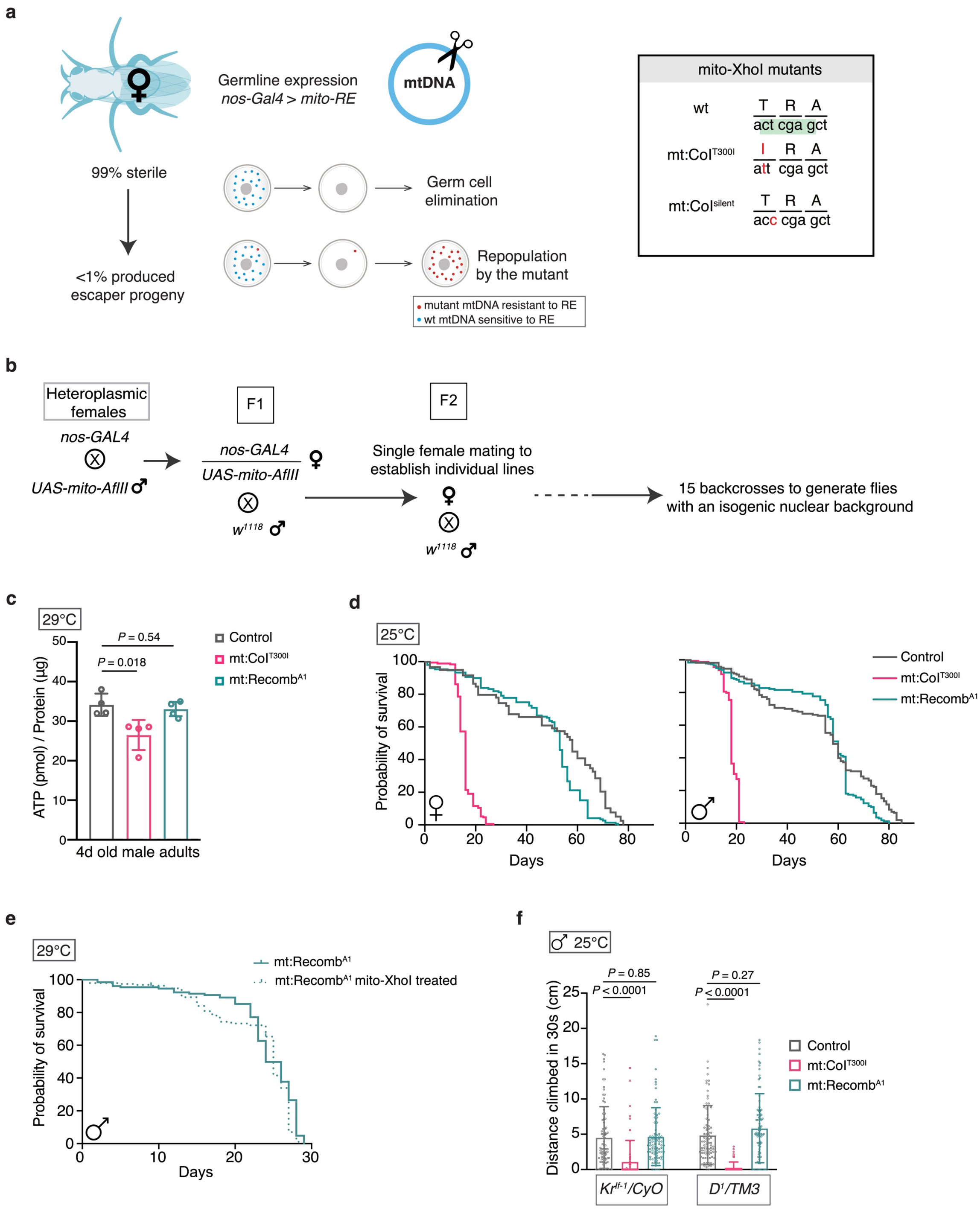

Supplementary Fig. 1: mtDNA recombination reveals intra-genome allelic interactions that rescue a homoplasmic mutant. a) A sketch illustrating the isolation of homoplasmic mtDNA mutants in *D. melanogaster* via the germline expression of a mito-RE that cuts the wild-type mtDNA once[1]. Two mutants used in this study, mt:CoI^{silent} and mt:CoI^{T300I}, were isolated in parallel by expressing mito-XhoI. They share the same mtDNA backbone as their mothers (*nos-GAL4*), except for the CoI^{T300} residue[2]. The recognition site of XhoI is highlighted in green. **b)** The crossing scheme to generate fly lines homoplasmic for recombinant mtDNA. *nos-GAL4* females heteroplasmic for *D. melanogaster* mt:CoI^{T300I} and *D. mauritiana* mtDNA were crossed with *UAS-mito-AflII* males to generate F1 females expressing mito-AflII in the germline. The F2 progeny were homoplasmic for a certain recombinant mtDNA genotype that lacked the AflII recognition site. Single-female crossings and backcrosses were performed to establish multiple mtDNA lineages with an isogenic nuclear background. **c)** Relative ATP levels in 4-day-old adult males shifted to 29°C after eclosure (n = 4 groups of 5 animals). Data represent mean ± SD (Source data), Student's *t*-test. **d)** Lifespan of flies homoplasmic for different mitochondrial genotypes at 25°C (n = 200 adult flies) (Source data). **e)** Lifespan of male flies with a rescuing recombinant mtDNA before and after mito-XhoI expression at 29°C (n = 200 adult flies) (Source data). **f)** The climbing ability of flies homoplasmic with a rescuing recombinant mtDNA in two different nuclear backgrounds (w^-*; Kr^{If-1}/CyO;* and w^-*;;D^1/TM3*, which replaces the 2nd and 3rd chromosomes of the original mt:Recomb^{A1} line, respectively). For each genotype, 12-day-old males raised and maintained at 25°C were used (n = 80 adult flies). Data represent mean ± SD (Source data), Student's *t*-test.

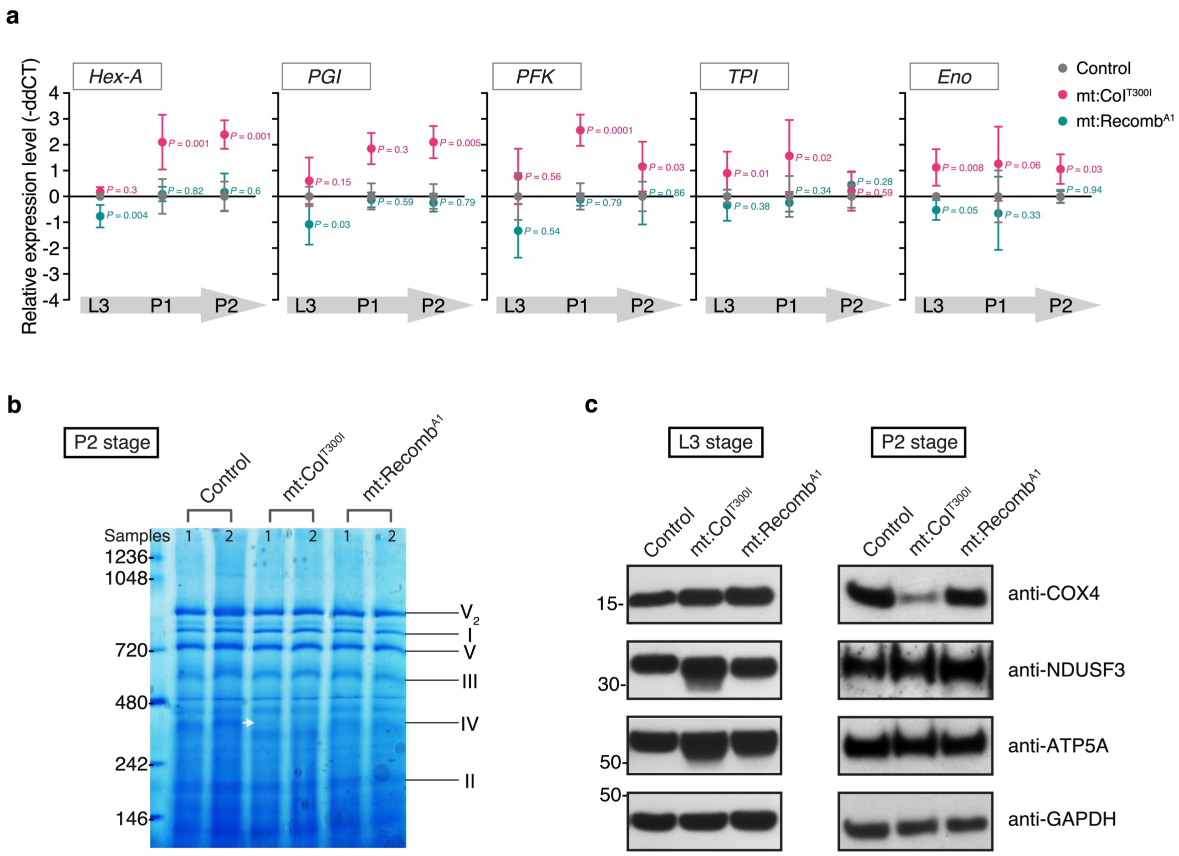

Supplementary Fig. 2: Complex IV level was reduced in the mt:CoI^{T300I} mutant at the pupal P2 stage. a) The expression level of key enzymes involved in glycolysis from larval to pupal stages measured by RT-qPCR (n = 4 groups of ten animals). Relative expression levels are represented by the differences in Ct values of RT-qPCR between genes of interest and the housekeeping gene *EF1α*. Data represent mean ± SD (Source data), Student's *t*-test. **b)** Coomassie blue staining of a BN-PAGE gel with digitonin-solubilised mitochondrial extracts isolated from P2 pupae. Two independent biological repeats for each mitochondrial genotype, labelled as 1 and 2, were examined. The complex bands were annotated based on our immunoblots and two previous studies probing *Drosophila* mitochondrial complexes by BN-PAGE and mass spectrometry (Shimada et al.[3] and Garcia et al.[4]). The arrow indicates a reduced complex IV signal in the mt:CoI^{T300I} mutant. The protein sizes are in kDa. **c)** Western blotting of complex I, IV, and V components of L3 larval and P2 pupal animals. Anti-NDUFS3, anti-COX4, and anti-ATP5A were used to blot complexes I, IV and V, respectively. GAPDH was used as a loading control. The protein sizes are in kDa. The representative blot of four immunoblotting experiments is presented here.

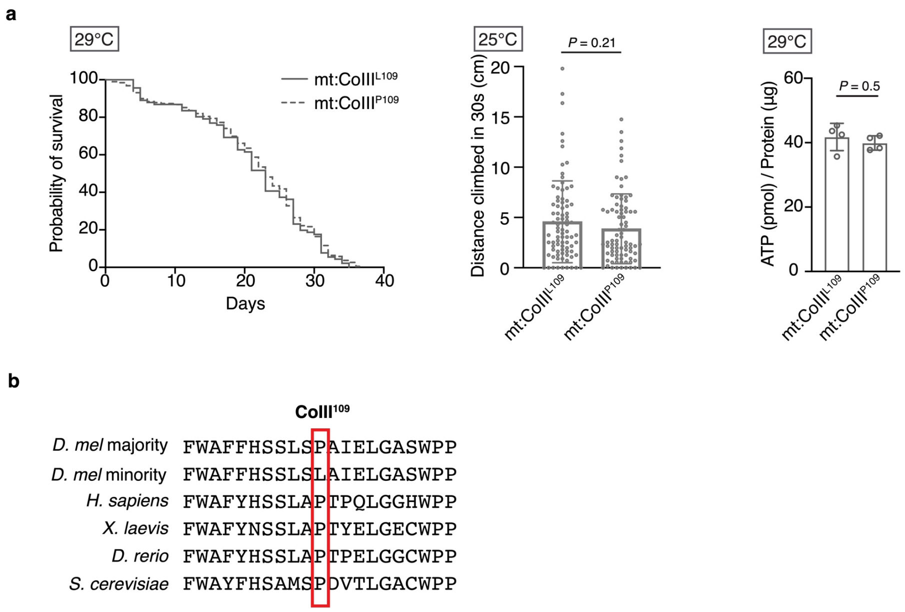

Supplementary Fig. 3: The proline versus leucine polymorphism at the CoIII^{109} residue per se had no impact on host fitness. **a)** The lifespan at 29°C (n = 200 adult flies), climbing ability (n = 80 adult flies) and ATP levels (n = 4 groups of 5 animals) of adult males homoplasmic with either mt:CoIII^{P109} or mt:CoIII^{L109}. For the climbing assay, 12-day-old males raised and maintained at 25°C were used. Data represent mean ± SD, Student's *t*-test. **b)** Partial protein alignment showing the CoIII^{109} residue (outlined by a red box) is proline for the reference genome of various species.

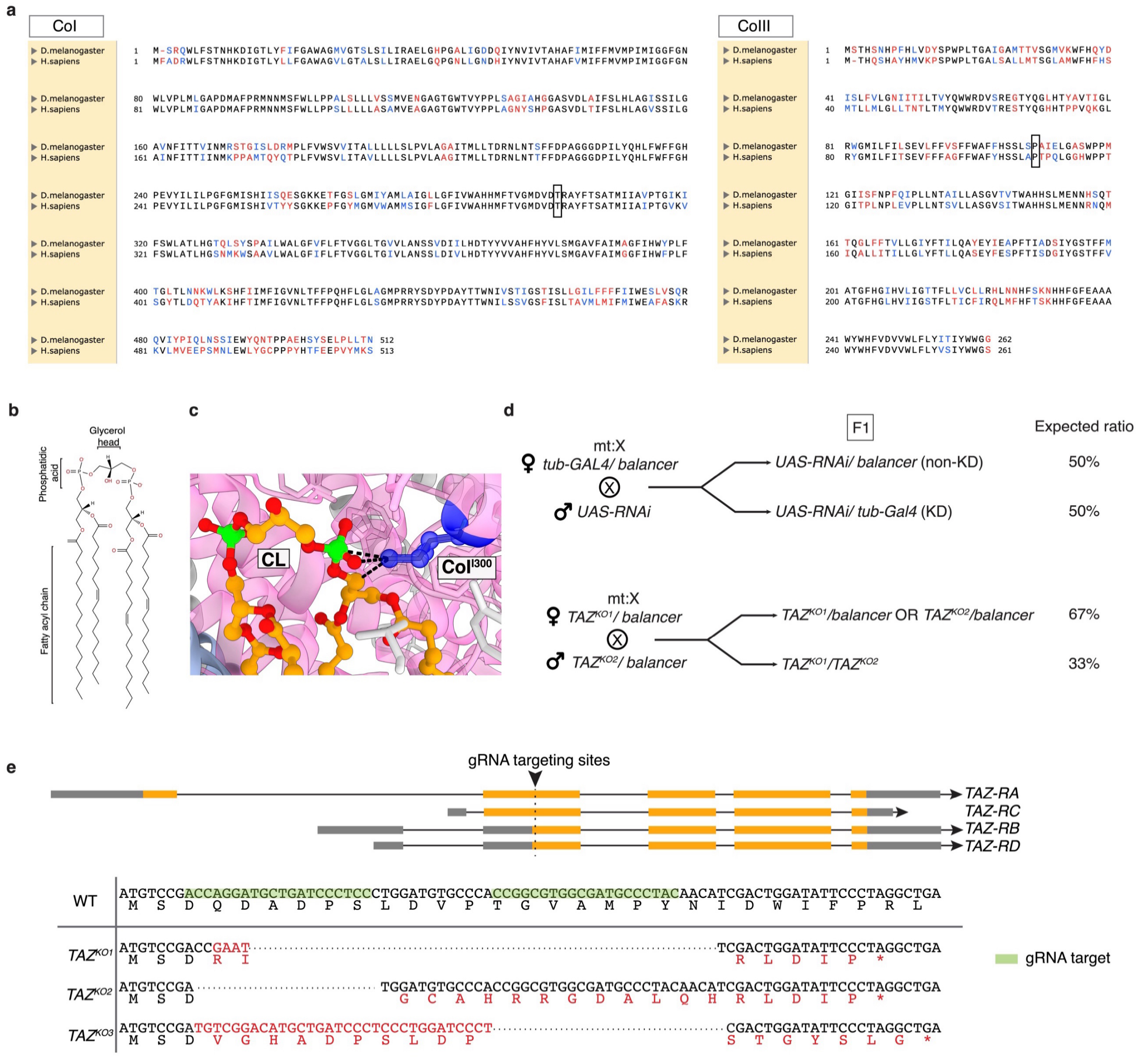

Supplementary Fig. 4: The mt:CoI^{T300I} mutant was sensitive to TAZ knockdown and knockout. a) The sequence alignment of *D. melanogaster* and *H. sapiens* CoI and CoIII proteins. The alignment was generated by the SnapGene software. The colours of amino acids indicate the level of difference: identical (black), blue (similar) and red (different). The CoI^{300} and CoIII^{109} residues are outlined by a black box. **b)** The structure of CL using CL 64:3 (CL 16:0_16:1_16:1_16:1) as an example. **c)** ChimericX modelling replacing threonine with isoleucine at the human CoI^{301} (fly CoI^{300} equivalent) residue revealed multiple clashes between the isoleucine and the CL headgroup. The simulation with isoleucine in a major rotameric state is shown here. The potential clashes are indicated by the dotted lines. **d)** The genetic crosses to generate flies with *TAZ* knockdown (KD) or knockout (KO) and the expected percentages of different progeny if knocking down or out *TAZ* does not affect viability. **e)** The sequences of *TAZ* KO mutants generated in this study. Top panel: *TAZ* isoforms, grey blocks (5' or 3' UTRs) and orange blocks (exons). Bottom panel: *TAZ* mutants, dotted lines (deletion), red nucleotides (insertion) and red amino acids (missense or nonsense mutations).

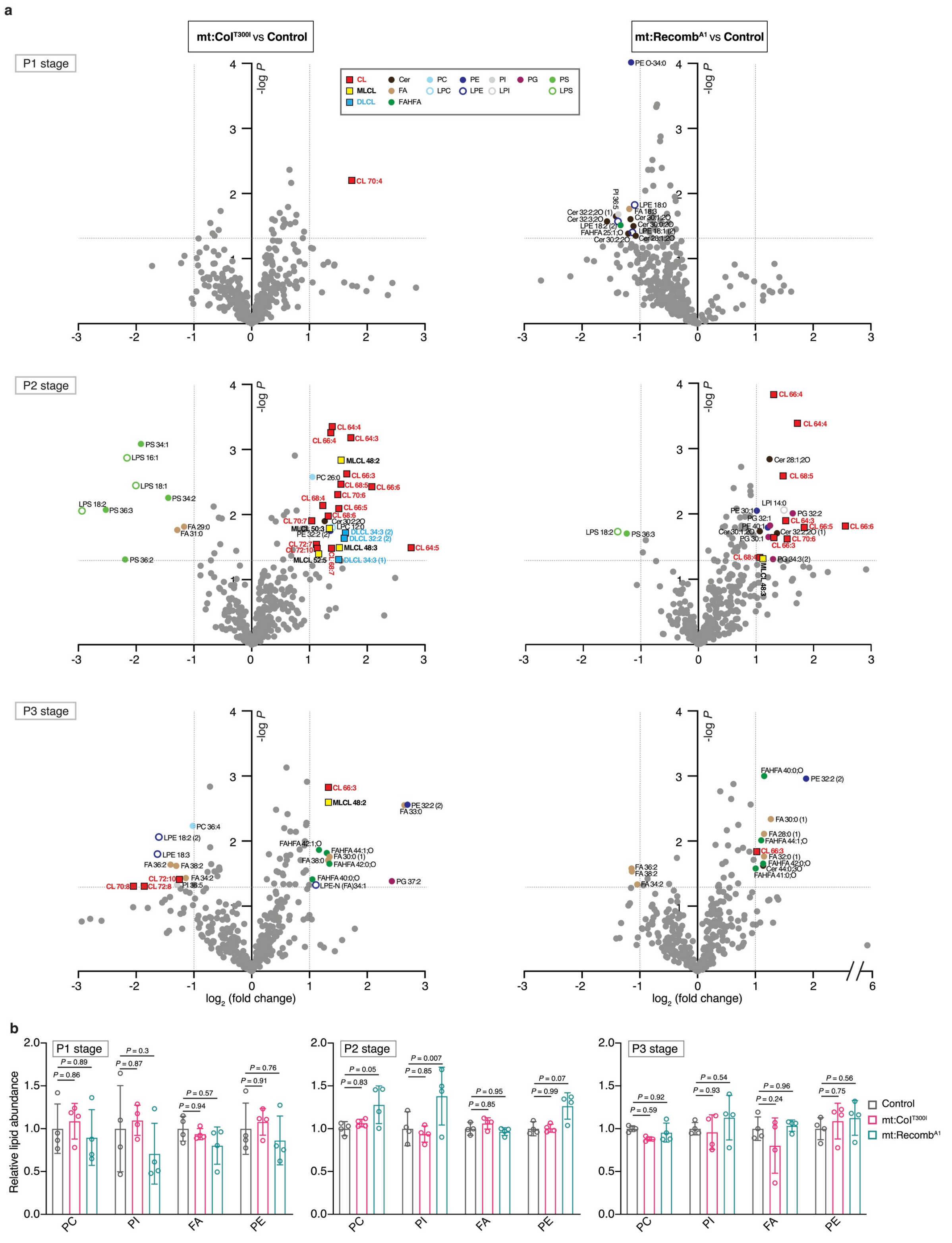

Supplementary Fig. 5: CL species of various acyl chain compositions were increased in mt:Col^{T300I} P2 stage animals. a) Volcano plots illustrating the identity of lipid species increased or decreased in mt:Col^{T300I} or mt:Recomb^{A1} compared to the control group at pupal stages (n= 4 groups of 50 animals). The *x*-axis represents $\log_2$ (fold change), and the *y*-axis represents $-\log_{10}$ (*P*-value). Each dot represents one lipid species. All lipid species with >2 fold change ($\log_2 x < -1$ or > 1) and $P < 0.05$ (-log P = 1.3, dotted line) are highlighted and labelled with names (Source data). See Supplementary Table 4 for all CL, MLCL and DLCL species measured by the lipidomics profiling. Welch's *t*-test. **b)** The total amount of fatty acids (FA), phosphatidylcholine (PC), phosphatidylethanolamine (PE), and phosphatidylinositol (PI) in mitochondria isolated from P1, P2 and P3 pupae (n = 4 groups of 50 animals). Different lipid species within the same group were summed for each genotype at the given developmental stage, and the relative amounts to the control group were plotted. Data represent mean ± SD (Source data), Student's *t*-test.

Supplementary Table 1: A list of *Drosophila* lines used in this study.

Line	Source
nos-GAL4 (III)	O'Farrell Lab, UCSF
nos-Cas9	Bloomington stock centre 54591
UAS-Dicer (III)	Bloomington stock centre 36510
tub-GAL4 (III)	O'Farrell Lab, UCSF
UAS-mito-AflII	Generated in this study
UAS-mito-NciI	This lab
$TAZ^{WKO.3\text{-}F11}$	Bloomington stock centre 82539
UAS-TAZ $RNAi^{JF1564}$	Bloomington stock centre 31009
UAS-TAZ $RNAi^{HMC03231}$	Bloomington stock centre 51484
Dp(2;3)GV-CH321-61C11 (*TAZ* duplication)	Bloomington stock centre 90556
Dp(3;2)GV-CH321-80E23 (*iPLA2* duplication)	Bloomington stock centre 90038
w^{1118}	This lab
mt:CoI^{T300I}	This lab
mt:CoI^{silent}	This lab
D. mauritiana (mt:mau)	This lab
w^{-}; $Kr^{If\text{-}1}$/*CyO;*	This lab
w^{-};;D^{1}/*TM3*	This lab

Supplementary Table 2: A list of primers used for Sanger sequencing *Drosophila* mtDNA

Primer	Sequence
mt186 Fwd	AAGCTACTGGGTTCATACCCC
mt984 Fwd	GGATTACCTCCATTTTTAGGATTTTTACC
mt1730 Fwd	GTGCTCCTGATATAGCATTCCCACG
mt2302 Fwd	GCTATTGGATTATTAGGATTTATTGT
mt3020 Fwd	GGCAGATTAGTGCAATAGATTTAAGCTC
mt4518 Fwd	CGACCTGGAACATTAGCTGTTCG
mt4807 Rev	AGCTCCTGTTAATGGTCATGGAC
mt5204 Fwd	CATTCACAAACTACTCAAGG
mt6115 Fwd	TCTTTAATTGAAGCCAAAAAGAGG
mt7214 Fwd	GCTTTAAATAAAGCATGAGTTAATAAATGA
mt7509 Rev	GCTGCTCCTACACCTGTTTCTGC
mt8468 Fwd	GGAGGAGCTGCTATATTAGCTG
mt8587 Rev	GTATCTTATGAACGTCTTGGAAGTCGAAG
mt9382 Fwd	CACAACCTAAAAAATAAGAAATTTCTGATC
mt10351 Fwd	GAAAATTCTTTATCTTTAAATAAATTATATAATTTTCC
mt10884 Fwd	GTAATAGGAACAGCTTTTATAGG
mt11517 Fwd	AGCTCGACCAGTTGAAGAACC
mt12822 Fwd	AACCAACCTGGCTTACACC
mt12923 Rev	GTTCAAATTTTAAGTCTGTTCGAC
mt13245 Fwd	CGTCCAACCATTCATTCCAGCC
mt14745 Rev	GTGCCAGCAGTCGCGGTTATAC

Supplementary Table 3: A list of primers used for RT-qPCR

Primer	Sequence
CoI RT-qPCR Fwd	GCTATTGGATTATTAGGATTTATTGT
CoI RT-qPCR Rev	TCCTAAAGCTCATAAAATAGCTGGAGA
CoIII RT-qPCR Fwd	CATTCACAAACTACTCAAGG
CoIII RT-qPCR Rev	GAGCTTCAATATATTCATAAGCTTG
Cox4 RT-qPCR Fwd	TGATGAACATCTTCGTGTACGAT
Cox4 RT-qPCR Rev	TTGATTTCCAGGTCGATGATG
TAZ RT-qPCR Fwd	CAGCCAGTTCGTCGTCTC
TAZ RT-qPCR Rev	GTTATCAGCTGGATCAGTCGCTC
iPLA2 RT-qPCR Fwd	GCAAGCCGGTCGATCTACATC
iPLA2 RT-qPCR Rev	GACCAAATGCGCGGAAATATGAG
CLS RT-qPCR Fwd	GTCGTGCCGTCCTCTCAC
CLS RT-qPCR Rev	CGTCGTGCGATCTGTCCATC
EF1α RT-qPCR Fwd	GCGTGGGTTTGTGATCAGTT
EF1α RT-qPCR Rev	GATCTTCTCCTTGCCCATCC
Hex-A RT-qPCR Fwd	TGTCAAGTGCTTCGTAACGTATGTG
Hex-A RT-qPCR Rev	GTGCTGCGGTATGGCATAGATG
PGI RT-qPCR Fwd	CGAACATGTTGGTGCTGTCAATG
PGI RT-qPCR Rev	GCCTCAAAAACTTTCACCACCCAG
PFK RT-qPCR Fwd	AGCGTCTGAACATCGTGATTGTG
PFK RT-qPCR Rev	TGGTTGCCGTCCAGAGAGAT
TPI RT-qPCR Fwd	ACCTCTTGAGCCTGATCGGG
TPI RT-qPCR Rev	AGAAGATCAAGGACTGGAAGAACGTG
EnO RT-qPCR Fwd	GATGCGCAGGATCTGGTTGTAC
EnO RT-qPCR Rev	GTGAGACCGAGGACTCGTTCATC

Supplementary Table 4: The CL species measured by LC-MS lipidomics

CL species	Average Mz
CL 64:3; CL 16:0_16:1_16:1_16:1	1345.9153
CL 64:4; CL 16:1_16:1_16:1_16:1	1343.8994
CL 64:5; CL 14:1_16:1_16:1_18:2	1341.8812
CL 66:3; CL 16:0_16:1_16:1_18:1	1373.9469
CL 66:4; CL 16:1_16:1_16:1_18:1	1371.9299
CL 66:5; CL 16:1_16:1_16:1_18:2	1369.9141
CL 66:6; CL 14:1_16:1_18:2_18:2	1367.8978
CL 68:4; CL 16:1_18:1_16:1_18:1	1399.9623
CL 68:5; CL 16:1_18:1_16:1_18:2	1397.9452
CL 68:6; CL 16:1_18:2_16:1_18:2	1395.9299
CL 68:7; CL 16:1_16:1_18:2_18:3	1393.9147
CL 70:3; CL 16:0_18:1_18:1_18:1	1430.0078
CL 70:4; CL 16:1_18:1_18:1_18:1	1427.9941
CL 70:6; CL 16:1_18:2_18:1_18:2	1423.9619
CL 70:7; CL 16:1_18:2_18:2_18:2	1421.9462
CL 70:8; CL 16:1_18:2_18:2_18:3	1419.9313
CL 72:3; CL 18:0_18:1_18:1_18:1	1458.0392
CL 72:4; CL 18:1_18:1_18:1_18:1	1456.0250
CL 72:7; CL 18:1_18:2_18:2_18:2	1449.9766
CL 72:8; CL 18:2_18:2_18:2_18:2	1447.9628
CL 72:10; CL 32:3_40:7	1443.9286
DLCL 32:2 (1); DLCL 16:1_16:1	871.4725
DLCL 32:2 (2);	871.4737
DLCL 34:2; DLCL 16:1_18:1	899.5045
DLCL 34:3 (1); DLCL 16:1_18:2	897.4889
DLCL 34:3 (2);	897.4894
DLCL 36:2; DLCL 18:1_18:1	927.5360
DLCL 36:4; DLCL 18:2_18:2	923.5045
DLCL 36:6;	919.4703
MLCL 48:0; MLCL 16:0_16:0_16:0	1113.7329
MLCL 48:2; MLCL 16:0_32:2	1109.7023
MLCL 48:3; MLCL 16:1_16:1_16:1	1107.6867
MLCL 50:3; MLCL 16:1_16:1_18:1	1135.7175
MLCL 50:4; MLCL 16:1_16:1_18:2	1133.7020
MLCL 52:5; MLCL 16:1_18:2_18:2	1159.7174
MLCL 54:3; MLCL 18:1_18:1_18:1	1191.7804

Supplementary References

1. Xu, H., DeLuca, S. Z. & O'Farrell, P. H. Manipulating the metazoan mitochondrial genome with targeted restriction enzymes. *Science* **321**, 575–577 (2008).

2. Ma, H., Xu, H. & O'Farrell, P. H. Transmission of mitochondrial mutations and action of purifying selection in *Drosophila melanogaster*. *Nat. Genet.* **46**, 393–397 (2014).

3. Shimada, S. *et al.* A unique respiratory adaptation in *Drosophila* independent of supercomplex formation. *Biochim. Biophys. Acta Bioenerg.* **1859**, 154–163 (2018).

4. Garcia, C. J., Khajeh, J., Coulanges, E., Chen, E. I.-J. & Owusu-Ansah, E. Regulation of mitochondrial complex I biogenesis in *Drosophila* flight muscles. *Cell Rep.* **20**, 264–278 (2017).
